# Regulation of the Tec family of non-receptor tyrosine kinases in cardiovascular disease

**DOI:** 10.1038/s41420-022-00927-4

**Published:** 2022-03-16

**Authors:** Zeyu Yin, Yuanming Zou, Dong Wang, Xinyue Huang, Shengjun Xiong, Liu Cao, Ying Zhang, Yingxian Sun, Naijin Zhang

**Affiliations:** 1 Department of Cardiology, the First Hospital of China Medical University, Shenyang, Liaoning China; 2 Key Laboratory of Medical Cell Biology, Ministry of Education; Institute of Translational Medicine, China Medical University; Liaoning Province Collaborative Innovation Center of Aging Related Disease Diagnosis and Treatment and Prevention, Shenyang, Liaoning China

**Keywords:** Medical research, Diseases, Biochemistry

## Abstract

Tyrosine phosphorylation by protein tyrosine kinases (PTKs) is a type of post-translational modification. Tec kinases, which are a subfamily of non-receptor PTKs, were originally discovered in the hematopoietic system and include five members: Tec, Btk, Itk/Emt/Tsk, Etk/Bmx, and Txk/Rlk. With the progression of modern research, certain members of the Tec family of kinases have been found to be expressed outside the hematopoietic system and are involved in the development and progression of a variety of diseases. The role of Tec family kinases in cardiovascular disease is receiving increasing attention. Tec kinases are involved in the occurrence and progression of ischemic heart disease, atherosclerosis, cardiac dysfunction associated with sepsis, atrial fibrillation, myocardial hypertrophy, coronary atherosclerotic heart disease, and myocardial infarction and post-myocardial. However, no reviews have comprehensively clarified the role of Tec kinases in the cardiovascular system. Therefore, this review summarizes research on the role of Tec kinases in cardiovascular disease, providing new insights into the prevention and treatment of cardiovascular disease.

## Introduction

Protein phosphorylation: refers to the process catalyzed by protein kinase to transfer the phosphate group of ATP to the substrate protein amino acid residues (serine, threonine, tyrosine), or bind GTP under the action of signal. It is a common regulation mode in organisms and plays an important role in the process of cellular signal transduction. Protein phosphorylation is the most basic, universal, and important mechanism for regulating and controlling protein viability and function. The main role of serine phosphorylation is to allosteric proteins to activate the activity of proteins, mainly referring to enzyme activity. In addition to allosteric and activating the activity of the protein, tyrosine phosphorylation can bind proteins to provide a structural gene to promote their interaction with other proteins to form multiprotein complexes. The formation of protein complexes further promotes the phosphorylation of proteins. Cycling, the signal generated by the initial protein phosphorylation turns on step by step. If a signal is initially generated that stimulates cell growth, this signal is eventually transferred to the nucleus, resulting in DNA replication and cell division. The enzymes that perform this modification are protein tyrosine kinases (PTKs), which catalyze the transfer of adenosine triphosphate to tyrosine residues on protein substrates [1]. Two classes of PTK exist in cells: transmembrane receptor PTKs and non-receptor PTKs [2].

Ninety unique tyrosine kinase genes have been identified in the human genome, of which 58 receptor PTKs are distributed in 20 subfamilies, and 32 non-receptor PTKs are distributed in 10 subfamilies [3, 4]. Their functions include cell growth, cell differentiation, and signal transmission. And it plays an important role in a variety of human diseases.

**Fig. 1:**
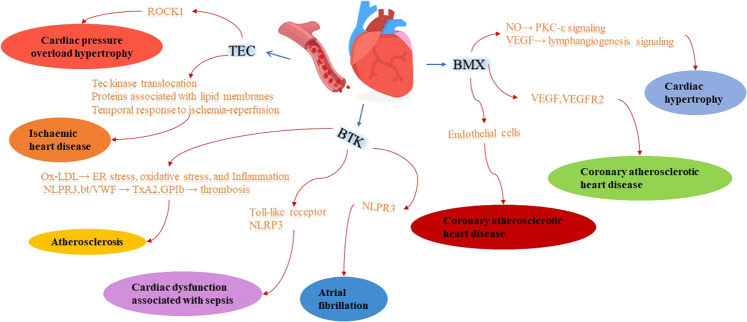
The regulation and functions of Tec family kinases in cardiac and vascular disease. Ox-LDL oxidized low-density lipoprotein, ROCK1 Rho kinase 1, ER endoplasmic reticulum, bt/VWF botrocetin/von Willebrand factor-mediated, TXA2 thromboxane A2, GPIb glycoprotein Ib, VEGF Vascular endothelial growth factor, VEGFR2 VEGF receptor 2, PKC-ε protein kinase C -ε.

Tec kinases, which are a subfamily of non-receptor PTKs represented by the first member, Tec, are key players in intracellular signaling in lymphocytes [5]. This family consists of five members: Tec, Btk, Itk/Emt/Tsk, Etk/Bmx, and Txk/Rlk. While Btk, Itk, and Txk are selectively expressed in hematopoietic cells, the expression patterns of Bmx and Tec are more widespread (Table 1). Many cells may express several Tec kinases, and it has been reported that they may functionally complement each other [6, 7]. PTK signaling plays an important role in the development of many diseases, including cancer, Alzheimer’s disease and diabetes mellitus, and the use of PTK inhibitors to treat disease is receiving increasing attention [8–10]. Cardiovascular system disease pathogenesis is complex, and multiple factors play a role in its development [11**–**13]. In recent years, much attention has been paid to cardiovascular disease and the role of PTKs in cardiovascular disease. At the same time Tec family kinases have also received more attention. Tec kinases are not only associated with immune system diseases and hematological diseases, but they are also involved in inflammatory diseases, infectious diseases, and cardiovascular diseases (Table 2) (Fig. 1). Some Tec kinase inhibitors have shown good application prospects in the treatment of cardiovascular disease (Table 3).

**Table 1:** Chromosome assignment and expression of Tec family kinases.

Kinase	Chromosome assignment	Expression
TEC	4p12	T cells, B cells
		Liver, heart, kidney and ovary
BTK	Xq22	T cells, B cells, innate immune cells, megakaryocytes and platelets
BMX	Xp22.2	Granulocytic monocyte lineage, endothelium of the endocardium and arteries
ITK	5q31–32	T cells, mast cells, natural killer cells
TXK	4p12	T cells

**Table 2:** The Tec family kinases functions and regulation.

Kinase	Mechanisms	Biological response	References
TEC	Ischemic injury as well as purines can stimulate Tec kinase translocation in cardiomyocytes. Tec regulates proteins associated with lipid membranes, and is involved in the temporal response of the heart to ischemia-reperfusion.	Ischaemic heart disease	[33, 34]
	Tec may play a cardioprotective role by binding to ROCK1.	Cardiac pressure overload hypertrophy	[33, 35]
BTK	BTK promotes ox-LDL-induced ER stress, oxidative stress, and inflammatory responses in macrophages, and BTK knockdown can inhibit ox-LDL-induced B signaling activation and inhibit M1 polarization in NK-κ macrophages.BTK regulates the NLRP3 inflammasome.Btk is involved in bt/VWF (Botrocetin/von Willebrand factor) -mediated lectin-induced TxA2 production and GPIb-dependent arterial thrombosis.	Atherosclerosis	[41–56]
	Btk plays a role in Toll-like receptor signaling and NLRP3 inflammasome activation.	Sepsis-related cardiac dysfunction	[46, 47, 57, 68]
	BTK promotes NLRP3 inflammasome activation.	Atrial fibrillation	[46, 47, 69]
BMX	The effect of Bmx on cardiac hypertrophy is mediated through the endothelial cells of the coronary vessels.	Cardiac hypertrophy	[73, 77]
	Bmx regulates VEGFR2 expression and VEGF-induced angiogenesis, and Bmx is also a downstream effector of VEGFR2. It is involved in ischemia-induced arteriogenesis and angiogenesis. Bmx is also involved in cell adhesion, migration and survival in TNF-induced angiogenesis.	Coronary atherosclerotic heart disease	[78–83]
	Bmx is involved in the mechanism of NO-induced PKC-ε signaling and VEGF-dependent lymphangiogenesis signaling.	Myocardial Infarction and Post-Myocardial Infarction	[84–87]
ITK	None	None	None
TXK	None	None	None

**Table 3:** So far, studies of Btk inhibitors related to cardiovascular disease.

Btk inhibitors	Disease	References
Acalabrutinib	Atherosclerosis	[53,55]
ONO/GS-4059	Atherosclerosis	[53,55]
Ibrutinib	Atherosclerosis	[53,55]
BGB-3111	Atherosclerosis	[55]
Evobrutinib	Atherosclerosis	[55]

## Structural features of Tec family kinases

**Fig. 2:**
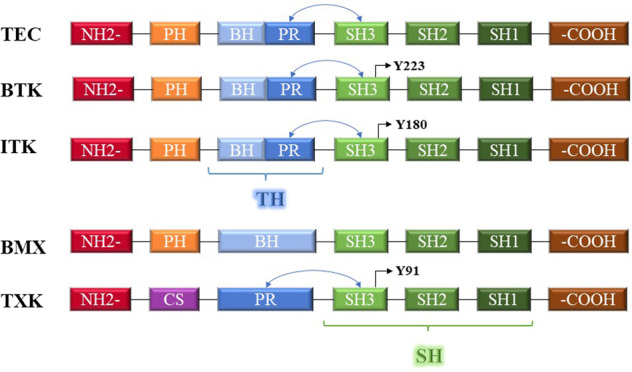
Schematic representation of Tec family kinases and their domains. PH pleckstrin homology, CS cysteine, BH Btk homology, PR proline-rich, TH Tec homology, SH Src homology, Y223, Y180, Y91 autophosphorylation sites; The PR motif can bind to the SH3 domain in an intramolecular manner.

Tec kinases have a highly conserved carboxy-terminal kinase domain and a relatively long and unique amino-terminal domain (Fig. 2). The amino-terminal domain can be further divided into a pleckstrin homology (PH) domain [14] and a Tec homology (TH) domain [15], which are characteristic of Tec kinases. The PH domain binds to phospholipids to mediate membrane association (the PH domain recruits Tec kinases to the cell membrane by binding certain phospholipids) [16], but the PH domain of Txk is replaced by a cysteine motif, which mediates membrane association by palmitoylation [17]. The TH domain includes an array of zinc-binding Btk homology (BH) motifs and one or two proline-rich (PR) motifs. The BH motif is involved in the binding site that constitutes the guanosine triphosphate-binding “active” Gα subunit of the heterotrimeric G protein. The PR motif can bind to the Src homology 3 (SH3) domain in an intramolecular manner, and this action may be important for protein targeting and enzyme activation [3]. Structurally, the TH domain of Txk lacks the BH sequence, the TH domain of Bmx lacks the PR sequence, and the SH3 domain shows a slightly different sequence. In all five kinases, the TH domain is followed by an SH3 domain and an SH2 domain [18]. The SH3 domain is primarily responsible for mediating protein–protein interactions [19, 20]. The SH3 domain of non-receptor PTKs appears to be implicated in the negative regulation of PTK activity [21]. SH2 can induce hyperphosphorylation of docking proteins [22], and it also stabilizes the intramolecular interactions between the SH3 domain and the PR motif [3]. SH2 is followed by a catalytic kinase domain, also known as the SH1 domain [23]. Tec family non-receptor PTKs are also unique in that they possess autophosphorylation sites inside their molecules. In studies on Btk, Itk, and Txk, all autophosphorylation sites were identified in the SH3 domain [24**–**27].

## Tec

### Tec function

Tec is very widely expressed in the human body and serves multiple functions. Tec tyrosine kinase plays an important role in the immune system which is involved in T cell development, cytokine production, T helper cell differentiation and B-cell development and activation [28, 29]. Tec, along with Btk, plays an important role in bone immunity and bone differentiation [30, 31]. Tec is also expressed in the liver, heart, kidney, and ovary [32], and is involved in the progression of a variety of diseases. Even in hepatoma cells, the expression of Tec has been found [32]. In recent years, the link between Tec and cardiovascular disease has attracted much attention, especially in ischemic heart disease and cardiac pressure overload hypertrophy.

### The role of Tec in ischemic heart disease

According to the World Health Organization, ischemic heart disease is caused by myocardial damage due to an imbalance between coronary blood flow and myocardial demand consequent of changes in the coronary circulation. The most common cause of ischemic heart disease is coronary artery stenosis and occlusion caused by coronary atherosclerosis. Insufficient blood supply and oxygen supply cause myocardial injury, which in turn develops into coronary heart disease or myocardial infarction. Tec translocates to Triton X-100 (TX-100)-insoluble fractions after ischemia and reperfusion. Adenosine and other purine nucleotides are released during ischemia-reperfusion. It has been proved that purinergic stimulation, but not hypoxia, induces translocation of Tec in cardiac cells [33]. Tec regulates proteins associated with lipid membranes. Tec is clearly involved in the temporal response of the heart to ischemia–reperfusion, and plays a protective role in myocardial ischemia [33]. HIF-1α, identified as a Tec interactor [33], is an important mediator of adenosine nucleoside-induced neuroprotection in hypoxia-stimulated PC12 cells [34]. Since Tec is activated by adenosine nucleotide stimulation in cardiomyocytes, it may participate in HIF-1α signaling during cardiac protection.

### The role of Tec in cardiac pressure overload hypertrophy

Rho kinase 1 (ROCK1), an immediate downstream kinase effector of RhoA GTPase, was be found as a Tec interactor [33]. A study [35] has indicated a critical role for ROCK1 in reactive fibrosis during pressure-overload hypertrophy. Tec may play a cardioprotective role by binding to ROCK1. However, the specifics of how Tec may contribute to this process are unclear.

## Btk

### Btk function

In 1993, Btk began to receive attention after it was reported that mutations in its gene result in X-linked agammaglobulinemia (XLA) in humans. Btk is the most extensively studied kinase among the Tec family of kinases. Btk plays an important role in immune function; is involved in many autoimmune diseases [36]. Btk primarily mediates B cell receptor signaling [37, 38]. Btk is also found in innate immune cells [38] and T cells [39] and is expressed in megakaryocytes and platelets [40]. Btk is involved in the inflammatory response in vivo and may play an important role at multiple points during the development and function of the bone marrow lineage. There is currently no definitive evidence that Btk is expressed in cardiomyocytes and vascular endothelial cells. What’s interesting is that Btk is closely related to a variety of cardiovascular diseases, such as atherosclerosis, cardiac dysfunction associated with sepsis and atrial fibrillation. And the mechanism of Btk in cardiovascular disease is complex.

### The role of Btk in atherosclerosis

Atherosclerosis is a chronic metabolic disease of the arterial wall characterized by lipid deposition and persistent aseptic inflammation. Atherosclerosis causes arterial stenosis, resulting in tissue and organ ischemia and hypoxia. Atherosclerosis is considered to underlie a variety of cardiovascular and cerebrovascular diseases. The effect of Btk on atherosclerosis manifests in the following three ways.

In the initial stages of atherosclerosis, reactive oxygen species signaling through Btk-p300-STAT1-PPARγ activates cholesterol crystal-induced CD36 expression and foam cell formation [41]. Btk promotes oxidized low-density lipoprotein (ox-LDL)-induced endoplasmic reticulum stress, oxidative stress, and the inflammatory response in macrophages, and Btk knockdown can inhibit ox-LDL-induced signaling activation and M1 polarization in NK-κ macrophages [42]. Btk can also promote atherosclerosis progression.

The NLRP3 inflammasome is an innate immune signaling complex. NLRP3 inflammasome activation helps drive atherosclerosis development and progression of vascular inflammatory responses and is also a major producer of cleaved interleukin (IL)-1 cytokines [43]. The NLRP3 inflammasome is activated by cholesterol crystals and is required for atherogenesis [44]. IL-1 cytokines are closely related to various cardiovascular diseases, such as atherosclerosis and myocardial infarction [45]. Btk is important for NLRP3 inflammasome activation. Btk regulates the assembly and, hence, activation of the NLRP3 inflammasome by binding to the ASC (apoptosis-associated speck-like protein containing a CARD) component [46, 47]. Inhibition of BTK by pharmacological or genetic means severely impairs activation of the NLRP3 inflammasome [47]. Targeting the NLRP3 inflammasome using Btk has received attention as another feasible approach for the treatment of cardiovascular disease [48].

Platelet activation and aggregation are key events in acute arterial thrombosis during plaque formation, which is one of the key factors affecting prognosis. Btk is involved in botrocetin/von Willebrand factor-mediated and lectin-induced TXA2 production and also in platelet membrane glycoprotein Ib-IX-V complex-dependent arterial thrombosis [49, 50]. Although Tec has been found to compensate for lack of Btk in patients with XLA leaving patients without a bleeding phenotype [51], Tec cannot compensate for the missing function of Btk during platelet activation and aggregation caused by atherosclerotic plaques [49]. Atherosclerotic plaques stimulate static platelet aggregation and platelet thrombosis through collagen I and III of the collagen receptor glycoprotein VI (GPVI). It has long been shown that Btk is important for collagen signaling through GPVI. GPVI is coupled to the Fc receptor gamma chain (FcRgamma). The FcRgamma-chain contains a consensus sequence known as the immune-receptor tyrosine-based activation motif (ITAM). Tyrosine phosphorylation of the ITAM upon GPVI stimulation is the initial step in the regulation of phospholipase C gamma2 (PLC*γ*2) isoforms via the tyrosine kinase p72 in platelets. Collagen and a collagen-related peptide (CRP) binds to GPVI and induced Btk tyrosine phosphorylation in platelets. Btk plays a crucial role during activation of platelets by CRP and collagen by regulating tyrosine phosphorylation and activation of PLC*γ*2 [52]. But Btk is not essential for thrombin-mediated platelet activation [52]; that is, Btk does not affect the physiological hemostatic process. Studies with multiple Btk inhibitors, such as acalabrutinib and ONO/GS-4059, have shown that Btk inhibitors specifically block the formation of platelet thrombi in atherosclerotic plaques, but do not affect physiological hemostasis [53**–**55]. Moreover, low-dose irreversible Btk inhibitors have unique potential as antiplatelet agents in atherothrombosis [56].

### The role of Btk in cardiac dysfunction associated with sepsis

Sepsis leads to a complex intramyocardial inflammatory response and sepsis-induced myocardial dysfunction [57, 58]. Toll-like receptor 3 (TLR3) mediates antiviral response by recognizing double-stranded RNA. Its cytoplasmic domain is tyrosine phosphorylated upon ligand binding and initiates downstream signaling via the adapter TIR-containing adapter inducing interferon-β (TRIF). Bruton’s tyrosine kinase phosphorylates Toll-like receptor 3 to initiate antiviral response [59], which facilitates recovery of cardiac dysfunction associated with sepsis.

The activation of NF-κB plays an important role in the cardiac dysfunction in sepsis [60, 61]. Inhibition of the activity of NF-κB attenuates the cardiac dysfunction in sepsis [62, 63]. Inhibition of BTK activity with ibrutinib or acalabrutinib reduces both the activation of NF-κB in septic hearts and the cardiac dysfunction caused by cecal ligation and puncture (CLP)-sepsis [64].

The activation of the NLRP3 inflammasome is a key component in the cardiac dysfunction [65] and the pathophysiology of sepsis [66]. This effect may result from activation of IL-1β and IL-18 via NLRP3 inflammasome [67, 68]. As previously stated BTK regulates the assembly and, hence, activation of the NLRP3 inflammasome by binding to the ASC component [46, 47]. Inhibition of BTK activity reduces both the assembly and subsequent activation of the NLRP3 inflammasome in septic hearts, resulting in reduced serum levels of IL-1β, and this contributes to the improvement of cardiac function [64].

### The role of Btk in atrial fibrillation

Atrial fibrillation (AF) is a type of arrhythmia caused by abnormal cardiac electrical conduction. However, an enhanced inflammatory response is frequently observed in AF patients. Btk promotes activation of the NLRP3 inflammasome as described above [46, 47]. Studies have shown that increases in NLRP3-inflammasome in cardiomyocytes might contribute to the evolution of atrial remodeling that promotes AF induction and maintenance. CM-specific activation of NLRP3 promotes abnormal sarcoplasmic reticulum (SR) Ca^2+^ release and electrical remodeling. Genetic inhibition of NLRP3 prevents spontaneous AF in CREM-transgenic mice (a well-characterized mouse model of spontaneous AF), which is sufficient to illustrate the importance of NLRP3 in atrial fibrillation [69]. But the role and mechanism of Btk in atrial fibrillation needs to be further studied.

**Fig. 3:**
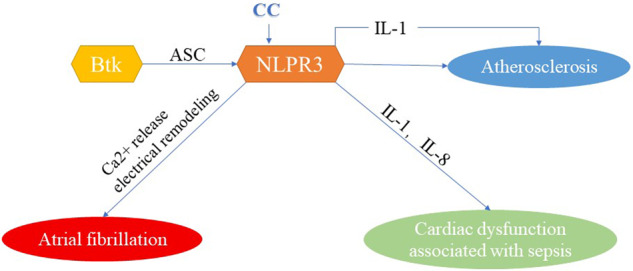
NLRP3-centered, cardiovascular disease network diagram related to Btk. CC cholesterol crystals, ASC apoptosis-associated speck-like protein containing a CARD, NLRP3 Human NACHT, LRR, and PYD domain-containing protein 3, IL interleukin.

Thus, through NLRP3 inflammasome, Btk is closely linked to atherosclerosis, cardiac dysfunction associated with sepsis, and atrial fibrillation. NLRP3 inflammasome is a hub where Btk is linked to cardiovascular disease (Fig. 3). Some Btk kinase inhibitors have shown good application prospects in the treatment of cardiovascular disease (Table 3).

## Bmx

### Bmx function

Bmx was initially found to be expressed in the granulocytic monocyte lineage within the hematopoietic system and is involved in the immune response. Bmx is also involved in certain inflammatory diseases, enhances IL-8 secretion, and is an important inflammatory mediator in vivo [70]. Bmx expression has also been demonstrated in a variety of cancers, such as prostate and breast cancer [71]. Bmx is also specifically expressed in the endothelium of the endocardium and arteries [72]; is involved in the growth, differentiation, apoptosis, and proliferation of epithelial cells; and plays an important role in cardiovascular disease.

### The role of Bmx in cardiac hypertrophy

The heart undergoes hypertrophy in response to hemodynamic overload to increase contractility and reduce ventricular wall stress. This adaptive hypertrophy will eventually transform into heart failure through pathological remodeling. Bmx knockout mice showed reduced cardiac hypertrophy in a transverse aortic constriction model [73] and reduced vascular endothelial growth factor-beta (VEGF-β) transgene-induced cardiac hypertrophy [74]. In an angiotensin II (Ang II)-induced cardiac hypertrophy model, the occurrence of cardiac hypertrophy was significantly reduced in Bmx-deficient or Bmx-inactivated mice. Bmx inactivation inhibits myocardial expression of genes associated with Ang II-induced inflammation and the extracellular matrix response, while in Bmx-inactivated hearts, Ang II administration maintains the expression of RNA encoding mitochondrial proteins [75]. Moreover, Bmx expression is high in arterial endothelial cells and minimal in cardiomyocytes, and Bmx RNA is detected in cardiac fibroblasts. These results strongly suggest that the effects of Bmx on cardiac hypertrophy are mediated through endothelial cells of the coronary vessels [75]. Mitochondria are particularly important for maintaining a healthy myocardium. STAT3 activation is an important cardioprotective factor associated with cardiac hypertrophy [76, 77], and the role of BMX gene silencing is to inhibit downstream STAT3 signaling [75].

### The role of Bmx in coronary atherosclerotic heart disease

Coronary atherosclerosis causes narrowing or obstruction of the vascular lumen, resulting in myocardial ischemia, hypoxia, or necrosis, causing coronary atherosclerotic heart disease. Long-term narrowing of the coronary arteries causes vascular remodeling to compensate for an insufficient blood supply to the heart. Vascular endothelial growth factor (VEGF) is an important proangiogenic factor [78]. Nuclear localization of Bmx mediates VEGF receptor 2 (VEGFR2) expression and regulates VEGF-induced angiogenesis [79]. Bmx is also a downstream effector of VEGFR2 [80] and plays an important role in ischemia-induced arteriogenesis and angiogenesis. Bmx is also involved in cell adhesion, migration, and survival in tumor necrosis factor-induced angiogenesis [81]. In a study using VEGF-2 gene therapy for coronary heart disease, patients had reduced angina pectoris and significantly improved perfusion and function [82]. It has also been shown that deletion of endothelial Bmx tyrosine kinase reduces tumor angiogenesis and growth [83]. Thus, Bmx may be a new target for the treatment of vascular diseases, such as coronary artery disease.

### The role of Bmx in myocardial infarction and post-myocardial infarction

Myocardial infarction is myocardial necrosis caused by acute or chronic, persistent ischemia and hypoxia of the coronary arteries. It can be complicated by ventricular aneurysm, cardiac rupture, mural thrombosis, arrhythmia, heart failure, cardiogenic shock, and post-myocardial infarction syndrome, which often endanger the patient’s life. Treatment and prognosis of myocardial infarction are equally important. Protein kinase C (PKC)-ε is a central modulator of cardioprotective signal transduction and can be used to prevent and treat myocardial infarction [84]. Moreover, Bmx plays a cardioprotective role by participating in nitric oxide-induced PKC-ε signaling [85]. Selective stimulation of cardiac lymphangiogenesis reduces myocardial edema and fibrosis and improves cardiac function after myocardial infarction [86]. Moreover, Bmx is involved in mediating the mechanism of VEGF-dependent lymphangiogenesis signaling [87]. Thus, the application of Bmx in the prevention, treatment, and prognosis of myocardial infarction demonstrates promise.

**Fig. 4:**
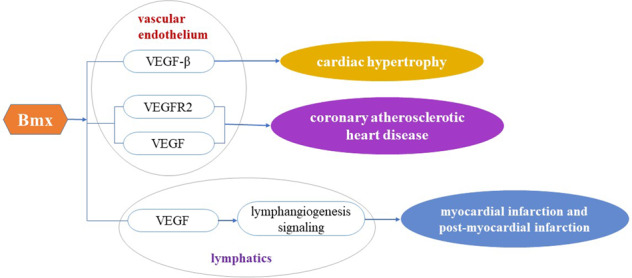
The link of Bmx to cardiovascular disease mediated through VEGF signaling. VEGF vascular endothelial growth factor, VEGF-β vascular endothelial growth factor-beta, VEGFR2 vascular endothelial growth factor receptor 2.

The role of Bmx in cardiac hypertrophy, atherosclerotic heart disease, and myocardiun infarction are all associated with VEGF signaling. Here we summarize the link of Bmx to cardiovascular disease mediated through VEGF signaling (Fig. 4).

## Itk and Txk

### Itk and Txk function

In T cells, four Tec kinases are expressed: Itk, Rlk, Tec, and Btk. These Tec kinases are important in T cell development and mature T cell differentiation, especially in TCR signaling [88]. Among them, Itk has the highest level of expression and is also known as Emt. It is expressed in mast cells [89] and natural killer cells [90] and is involved in certain autoimmune diseases, such as asthma [91] and atopic dermatitis [92]. Itk is also associated with inflammatory reactions [93], certain cancers [94] and viral infection [95]. Itk inhibitors have achieved beneficial results in many studies on disease treatment [96, 97]. Txk, also known as Rlk, is mainly detected in T cells and some myeloid cell lineages [98]. Txk has been implicated in immune-inflammatory diseases, such as rheumatoid arthritis, bronchial asthma, and atopic dermatitis [99, 100]. However, no association between Itk and Txk and cardiovascular disease has been found thus far.

## Conclusions and prospects

Tec is a stress-activated kinase. Upon cardiac ischemic injury, Tec kinase is translocated and exerts cardioprotective effects by eliciting downstream signaling through binding to H1F-1α and ROCK1. Tec kinases are functionally diverse and expressed in a variety of organs, with clear evidence of expression in the heart. Tec kinase plays an important role in the growth and development of immune cells. If the expression of Tec gene is inhibited in the heart, will it have an effect on the function of the heart? Currently, only a clear association between Tec and Ischemic heart disease was found in the current study. So it is unknown whether Tec has a corresponding relationship with other cardiovascular diseases and whether it also plays a protective role in it.

Btk promotes the progression of atherosclerosis by participating in the Btk–p300–STAT1–PPARγ pathway and promoting a series of responses induced by ox-LDL and NLRP3. Btk promotes platelet activation mediated by atherosclerotic plaques. Although Btk initiates an antiviral response by activating TLR3, Btk aggravates the cardiac dysfunction associated with sepsis by promoting the conduction of NF-κB and NLRP3 and their downstream signals. Btk contributes to the development of atrial fibrillation by affecting NLRP3. Many studies have demonstrated that Bmx can reduce the occurrence of cardiac hypertrophy, acting through the coronary endothelium and STAT3. Overall, Btk inhibitors have a good application prospect in the treatment of Atherosclerosis and cardiac dysfunction associated with sepsis, providing us with new ideas for cardiovascular disease treatment. However, it is unknown whether Btk inhibitors can be used in the clinical treatment of cardiovascular disease and whether they can have the expectant effect of treatment or prevention. A large number of studies are needed to explore the clinical significance of Btk inhibitors. Interestingly, while there is no evidence for Btk expression in the heart and blood vessels, we can see that through NLRP3, Btk is closely linked to three diseases: Atherosclerosis, Cardiac dysfunction associated with sepsis, and Atrial fibrillation. Are NLRP3 involved in other cardiovascular diseases? Are Btk involved in other cardiovascular diseases via NLRP3? We also need to further explore and clarify the interaction mechanism of Btk with NLRP3.

Bmx knockout can reduced VEGF-β transgene-induced cardiac hypertrophy. Mitochondria are particularly important for maintaining a healthy myocardium. The role of BMX gene silencing is to inhibit downstream STAT3 signaling which is an important cardioprotective factor associated with cardiac hypertrophy. Bmx exhibits positive and negative effects in cardiac hypertrophy. If the expression of Bmx is inhibited, it still needs to be clarified how it will eventually have an effect on cardiac hypertrophy. Bmx is involved in VEGF-induced angiogenesis and plays a beneficial role in coronary atherosclerotic heart disease. Bmx protects the heart in myocardial infarction by participating in nitric oxide-evoked PKC-ε signaling, which is involved in mediating VEGF-dependent lymphopoiesis signaling. We note that the effect of Bmx on cardiovascular disease, is mediated through VEGF signaling, which is very similar to the link between Btk and cardiovascular disease. VEGF signaling is associated with a variety of cardiovascular diseases, then it is unknown whether Bmx is involved in other cardiovascular diseases through VEGF signaling.

The expression of Tec kinases is widespread. In recent years, the association of Tec kinases, especially Btk and Bmx, with cardiovascular diseases has received extensive attention. To explore the relationship between Tec family tyrosine kinases and cardiovascular diseases, would provide us with new ideas for cardiovascular disease treatment.

## Supplementary information

Instructions for Authors

## Data Availability

The [DATA TYPE] data used to support the findings of this study are available from the corresponding author upon request.
