# Supplementary material for: Regulation of the Tec family of non-receptor tyrosine kinases in cardiovascular disease
Source: Cell Death Discov. 2022 Mar 16;8:119. doi: 10.1038/s41420-022-00927-4 (PMC8927484; doi:10.1038/s41420-022-00927-4)
Supplement: Supplementary file 1 — Instructions for Authors [file 41420_2022_927_MOESM1_ESM.docx]

Since three corresponding authors cannot be filled in simultaneously in the computer system, it is hereby explained to the editor that Zhang Naijin，Yingxian Sun，Ying Zhang are corresponding authors.
